# Does Primary Vomer Flap Significantly Affect Maxillary Growth?

**DOI:** 10.29252/wjps.9.1.62

**Published:** 2020-01

**Authors:** Abolhasan Emami, Haleh Hashemzadeh

**Affiliations:** 1 Department of Plastic Surgery, Iran University of Medical Sciences, Tehran, Iran;; 2 Department of Orthodontics, School of Dentistry, Tehran University of Medical Sciences, Tehran, Iran

**Keywords:** Cleft palate, Vomer flap, Maxillary growth

## Abstract

**BACKGROUND:**

Cleft lip and palate (CLP) is a common congenital anomaly. Efficient surgical management of CLP is challenging in severe cases with wide clefts. Use of primary vomer flap simultaneous with cleft lip repair is effective in some cases, but remains a challenging topic.

**METHODS:**

This study evaluated 81 non-syndromic CLP patients with extensive palatal cleft and no other underlying condition. Thirty-nine patients (group A) who were infants over 6 months of age underwent primary vomer flap during lip repair to decrease the size of their extensive palatal cleft. The results in this group were compared with group B (n=42) who did not receive primary vomer flap.

**RESULTS:**

Comparison of the two groups showed that although maxillary growth impairment and maxillary constriction had a higher frequency in group A, the palatal cleft was smaller among them, which enabled easier and more efficient cleft repair in the next step. The difference in maxillary growth impairment was not significant between the two groups. However, the prevalence of some complications such as velopharyngeal incompetence and maxillary growth impairment was slightly higher in group A compared with group B.

**CONCLUSION:**

Use of primary vomer flap at the time of lip repair can decrease the size of palatal cleft and enhance its later closure. However, since impairment of the maxillary growth was slightly (but insignificantly) higher in the vomer flap group, it should be performed at ages over 6 months of age, as well as in certain cases.

## INTRODUCTION

Cleft lip and palate (CLP) is among the most common congenital anomalies in infants.^1^^-^^3^ The parents of CLP patients are often in demand of immediate repair of the cleft lip because in the Iranian culture, as in many other countries worldwide, CLP is considered as a defect with significant psychological defect with huge psychological impacts on the family. Repair of the cleft palate ranks second in terms of significance to the parents after repair of the cleft lip, because creating a normal lip contour with no or minimal scarring is the ultimate demand of the parents.^2^^-^^6^

However, cleft palate compromises the physiological functions such as deglutition, and is associated with ear problems, speech problems, and eventual impairment of the growth of the maxilla and midface.^2^^-^^6^ In many cases, the parents insist on surgical correction of the cleft because they do not want others to find out about the congenital defect of their newborn. In some patients, CLP is part of a syndrome and is associated with some other congenital anomalies. Also, CLP has several subtypes and may vary in size from a micro-type cleft lip to complete CLP. 

Treatment planning is a critical step in management of patients with wide CLP. Until two or three decades ago, lip adhesion used to be the method of choice for management of wide cleft lips. This technique would successfully convert a wide cleft to an incomplete cleft and then the final repair would be performed.^7^^,^^8^ Following the advent of naso-alveolar molding and taping, the majority of CLPs, even wide cleft lips, are now definitively repaired in the first phase. A preliminary approach for management of wide CLP cases is to use primary vomer flap followed by primary repair of the cleft lip in order to close or narrow the wide cleft palate and convert it to a smaller cleft for easier closure in the next phase. 

In other words, this approach aims to convert a complete cleft to a narrower cleft in unilateral cases. In bilateral cases, this approach aims to close the cleft palate at the wider side and convert the case to a unilateral CLP. However, a major concern with regard to the use of primary vomer flap is the risk of impairment of growth and development of the midface in these patients compared with controls. The results of studies on this topic are controversial, and some authors have completely refuted its application,^7^ while some other still use it.^8^ The purpose of this study was to compare the results of primary vomer flap approach with conventional management of CLP patients. 

## MATERIALS AND METHODS

This retrospective descriptive, cross-sectional study evaluated CLP patients presenting to Saint Fatima Hospital, a referral center for plastic and reconstructive surgery, from 2005 to 2015 who were operated by the senior author of this paper and were followed-up. During this period, the majority of syndromic and non-syndromic CLP patients with wide CLPs are referred to to Saint Fatima Hospital. The patients with incomplete or narrow clefts are often managed in other hospitals. The inclusion criteria were CLP patients with wide cleft. 

The exclusion criteria were cases with incomplete clefts, cleft lip alone, syndromic CLP, and underlying systemic conditions, such as cardiac diseases. Patients who did not show-up for the follow-ups were excluded as well. Patients with premature labor were excluded. The two groups were matched in terms of nutrition (breastfeeding and formula). Also, all patients were matched in terms of weight and growth indices by a pediatrician and were within the normal growth curve. 

The inclusion criteria were optimal conditions to undergo surgery in terms of age, weight and paraclinical parameters. The rule of tens was used for lip surgery (minimum age of 10 weeks, minimum weight of 10 lbs. and minimum hemoglobin concentration of 10 mg/dl in both groups). 

A total of 81 patients met the eligibility criteria, including 45 females and 36 males. The patients were divided into two groups of A (vomer flap approach) and B (conventional approach). In group A, the parents of patients assigned to this group declared their consent to our treatment plan after receiving comprehensive information about the procedure. 

In these patients (n=39), the primary vomer flap was performed in the first session. The surgical procedure was performed after the 6 months of age. In group B, the parents of patients in this group (n=42) did not want to postpone the surgical procedure and insisted on the conduction of lip closure surgery at the earliest time possible. The parents of patients in group A signed informed consent forms prior to surgery. Patients in group B underwent the conventional surgical procedure after obtaining written informed consent from the parents approved by IRB and HIPAA compliant. 

In group A, the surgical procedure was performed after 6 months of age and the cleft lip closure was performed along with the primary vomer flap. In this group, three patients had isolated cleft palate in whom, the primary vomer flap was performed unilaterally to close the wide gap. The mean age of patients at the time of lip surgery was 5 months in both groups. The mean age of patients were 10 and 17 months at the time of soft palate and hard palate surgery, respectively. The mean duration of follow-up was 6.5 years (range 4 to 10 years). 

All patients were under supervision of the cleft team that included a pediatrician, an orthodontist, a speech therapist, an ear-nose-throat specialist, a social worker, nurses and residents, who were all supervised by the senior author. SPSS software was used for statistical analysis and Chi-Square test for comparison of variables. A p value less than 0.05 was considered statistically significant.

## RESULTS

Regarding the type of CLP in study patients, unilateral cleft lip and palate was seen in 29 and 33 patients of group A and B, respectively. Bilateral cleft lip and palate was visible in 7 patients of group A and 8 patients of group B. Cleft palate alone was noted in 3 and 1 patients of group A and B, respectively. Table 1 illustrated the complications following the completions of surgical procedures in the two groups. Table 2 demonstrated the type of maxillary growth problems according to the opinion of an orthodontist. 

**Table 1:** Complications following the completion of surgical procedures in the two groups

**Complications**	**Group A**	**Percentage**	**Group B**	**Percentage**
Fistula	3	7.6	4	9.5
Partial necrosis of palatal flap	0	0	1	2.3
Complete dehiscence of palatal repair	0	0	1	2.3
Hypernasality	4	10.2	5	11.9
Velopharyngeal incompetence	3	7.6	2	4.7
Maxillary growth retardation	7	17.9	3	1.7

**Table 2:** Type of maxillary growth problems according to the opinion of the orthodontist

**Type of problem**	**Group A**	**Percentage (n=39)**	**Group B**	**Percentage (n=42)**
Contracted maxilla	2	5.1	1	2.3
Maxillary retrusion	5	12.8	2	4.7
Cross bite	1	2.5	1	2.3

**Fig. 1:**
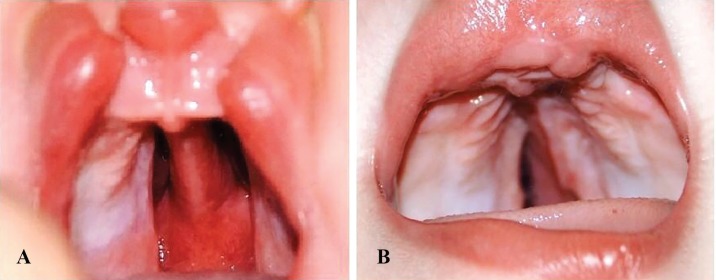
**A: **A case of bilateral cleft lip and palate before operation. **B: **post-operative view of the same case after lip repair and simultaneous primary vomer flap for closure of left side cleft palate (wider side) to facilitate the further palatoplasty for precise repair of the hard and soft palate

The prevalence of maxillary growth impairment was insignificantly higher in patients who underwent primary vomer flap surgery compared with the control group (Chi-square test, *p*>0.05). The complementary surgical procedures performed for patients during the follow-up period included pharyngeal flap in five patients, fistula repair in four patients, redo palatoplasty in one patient, repair of partial necrosis in one patient and alveolar bone grafting in three patients. In our study, the primary vomer flap surgery enhanced the closure of wide cleft palates (Figure 1). However, the follow-up results for an average period of 5.5 years revealed that the prevalence of maxillary growth impairment was higher in patients who underwent primary vomer flap surgery compared with the control group (*p*>0.05). 

## DISCUSSION

Despite the availability of different successful approaches for cleft palate repair, long-term results often indicate impaired growth of the midface and maxilla in a percentage of patients.^8^^-^^10^ On the other hand, studies on CLP patient populations that did not undergo surgical correction of their cleft until adulthood have shown complete growth and development of the midface and normal skeletal cephalometric relationships.^11^^,^^12^ A correlation has been confirmed between CLP repair and midface retrusion as surgical repair of CLP, irrespective of the adopted technique, results in scar tissue formation at the middle of the palate, which can impair growth in the sagittal plane and result in a contracted maxillary arch.^13^^-^^15^

A major concern with regard to the use of primary vomer flap is the risk of impairment in growth and development of the midface in of patients compared with conventionally managed controls.From 1975 to 1977, several cases of midface growth impairment were reported by Friede and Johanson.^2^ The popularity of vomer flap approach decreased afterwards; however, it never became obsolete and a number of contemporary surgeons still use the vomer flap approach. It was never confirmed that the vomer flap approach would cause a significant growth impairment.

We all know that leaving a CLP open for a long period of time can result in over-growth of the midface components such as the vomer, maxilla and premaxilla. Previously, in some cases, we had to resect the excess, overgrown vomer bone and even premaxilla in order to be able to close the cleft; these patients often developed retrusion of the premaxilla during the puberty and adulthood. However, Maggiulli *et al.* in 2014 showed that the maxillary dental arch in patients who had undergone vomer flap surgery was smaller than that in patients who had not undergone this surgical procedure, and this difference was statistically significant. But, the differences in other maxillary dimensions and the palate were not significant.^1^

In our study, the primary vomer flap surgery enhanced the closure of wide cleft palates (Figure 1). However, the follow-up results for an average period of 5.5 years revealed that the prevalence of maxillary growth impairment was higher in patients who underwent primary vomer flap surgery compared with the control group (*p*>0.05). Although the two groups were not significantly different regarding other outcomes and complications, it seems that the primary contribution of primary vomer flap surgery does not worth the occurrence of major growth impairment of the maxilla. However, further studies with larger sample sizes and longer follow-ups are required to cast a final judgment in this respect. 

Our results indicated that the primary vomer flap approach for management of CLP patients effectively closed or decreased the size of wide cleft palate. However, in some cases, this was achieved at the cost of contraction of the maxillary arch and its growth impairment. Thus, the primary vomer flap approach is suggested for use in selective patients. Further studies with longer follow-ups may provide more information in this regard.
